# Genome-Wide Identification and Evolution Analysis of Trehalose-6-Phosphate Synthase Gene Family in *Nelumbo nucifera*

**DOI:** 10.3389/fpls.2016.01445

**Published:** 2016-09-29

**Authors:** Qijiang Jin, Xin Hu, Xin Li, Bei Wang, Yanjie Wang, Hongwei Jiang, Neil Mattson, Yingchun Xu

**Affiliations:** ^1^College of Horticulture, Nanjing Agricultural UniversityNanjing, China; ^2^Leisure Agricultural Section, Institute of Agricultural Science of Taihu Lake DistrictSuzhou, China; ^3^Horticulture Section, School of Integrative Plant Science, Cornell UniversityIthaca, NY, USA

**Keywords:** trehalose-6-phosphate synthase, *Nelumbo nucifera*, protein properties, molecular evolution, expression pattern

## Abstract

Trehalose-6-phosphate synthase (TPS) plays a key role in plant carbohydrate metabolism and the perception of carbohydrate availability. In the present work, the publicly available *Nelumbo nucifera* (lotus) genome sequence database was analyzed which led to identification of nine lotus *TPS* genes (*NnTPS*). It was found that at least two introns are included in the coding sequences of *NnTPS* genes. When the motif compositions were analyzed we found that *NnTPS* generally shared the similar motifs, implying that they have similar functions. The *d*_*N*_/*d*_*S*_ ratios were always less than 1 for different domains and regions outside domains, suggesting purifying selection on the lotus *TPS* gene family. The regions outside TPS domain evolved relatively faster than NnTPS domains. A phylogenetic tree was constructed using all predicted coding sequences of lotus *TPS* genes, together with those from *Arabidopsis*, poplar, soybean, and rice. The result indicated that those *TPS* genes could be clearly divided into two main subfamilies (I-II), where each subfamily could be further divided into 2 (I) and 5 (II) subgroups. Analyses of divergence and adaptive evolution show that purifying selection may have been the main force driving evolution of plant *TPS* genes. Some of the critical sites that contributed to divergence may have been under positive selection. Transcriptome data analysis revealed that most *NnTPS* genes were predominantly expressed in sink tissues. Expression pattern of *NnTPS* genes under copper and submergence stress indicated that NNU_014679 and NNU_022788 might play important roles in lotus energy metabolism and participate in stress response. Our results can facilitate further functional studies of *TPS* genes in lotus.

## Introduction

Trehalose, known as a non-reducing disaccharide of glucose, has been found in wide range of diverse organisms, for example, bacteria, fungi, invertebrates, and plants (Lyu et al., 2013). In higher plants, trehalose accumulates at quite low concentrations, with the exception of resurrection plants (Paul et al., 2008). It was found that in trehalose-rich organisms, trehalose functions both as a carbon source and as an osmoprotectant such in certain microorganisms and insects (Elbein et al., 2003; Zang et al., 2011). There is crucial evidence that trehalose helps prevent heat, osmotic, nutrient, dehydration stress, and toxic chemicals from damaging organisms (Chary et al., 2008). Trehalose synthesis in plants only occurs via the TPS/TPP pathway (Avonce et al., 2006; Jiang et al., 2010). In the first step of this pathway, trehalose-6-phosphate synthase (TPS) joins uridine diphosphoglucose with glucose 6-phosphate, thus forming trehalose 6-phosphate (T6P; Jiang et al., 2010). Then, T6P is dephosphorylated as catalyzed by trehalose-6-phosphate phosphatase (TPP) to produce the disaccharide trehalose. TPS expression levels in cotton increased under drought stress (Mu et al., 2016). When maize was under salt and temperature stress, *TPS* genes were upregulated (Jiang et al., 2010). Genes which controlled the production of enzymes of trehalose synthesis, including the *AtTPS1* gene from *Arabidopsis thaliana* (Blázquez et al., 1998; Jiang et al., 2010). These findings made it practical to attain transgenic plants altered by a *TPS* gene of plant origin. Most transgenic plants with overexpressed TPS and/or TPP genes exhibited great tolerance to abiotic stress (Garg et al., 2002; Jang et al., 2003; Karim et al., 2007; Ge et al., 2008).

Although numerous studies found that over-expressing *TPS* and *TPP* genes could improve abiotic stress tolerance (Holmström et al., 1996; Jang et al., 2003; Schluepmann et al., 2004; Jiang et al., 2010), observed stress tolerance of transgenic lines did not correlate with trehalose amount (van Dijken et al., 2004). Researchers have investigated function of plant trehalose biosynthetic enzymes by identifying mutations in *Arabidopsis TPS1* gene and seeing which were still effective in recovering homologous gene function for yeast mutants deficient in TPS functions (Blázquez et al., 1998; Müller et al., 2001; Vogel et al., 2001). Beyond these stress response, recent intriguing evidence has implicated that *TPS* genes are important modulators in plant development and inflorescence architecture (Satoh-Nagasawa et al., 2006).

The plant *TPS* gene family belongs to a small gene family with multiple copies. Members of this gene family showed widespread functional diversification (Lunn, 2007; Chary et al., 2008; Vandesteene et al., 2010; Yang et al., 2012). The *Arabidopsis TPS* gene family contains 11 members (*AtTPS1*-*11*; Shima et al., 2007); rice also contains 11 members (*OsTPS1-11*; Tuskan, 2006; Vandesteene et al., 2010), and poplar contains 12 members (*PtTPS1-12*; Vandesteene et al., 2010). The TPS family is divided into two distinct clades, class I and class II, and this dichotomy occurred early in the evolution of green plants (Jang et al., 2003; Shima et al., 2007; Suzuki et al., 2008; Ramon et al., 2009). Apart from polyploid and allopolyploid species, *Arabidopsis* is quite uncommon among all the angiosperms which have large number class I *TPS* genes (Delorge et al., 2015). Particularly, distinct characteristics of class I and class II *TPS* genes are displayed in gene expression patterns, copy number, physiological functions, and enzyme activity.

Sacred lotus (*Nelumbo nucifera*, Gaertn.) is a basal eudicot which is of significant cultural, agricultural, medicinal, as well as religious importance. TPS plays important role in plant response to environmental stimuli, and thus the study of TPS would be very important for lotus breeding and the research of stress-resistance mechanism in lotus. In this study, we investigated the lotus *TPS* genes from whole genome-wide studies and their evolutionary relationship with other *TPS* genes. We explored TPS gene family members' expression patterns in various tissues and in response to various stresses. Having conducted a thorough analysis of gene sequences, molecular evolution, gene structures, and gene expression patterns, we offer an effective framework for further functional characterization of lotus *TPS* gene families.

## Methods

### Identification of lotus *TPS* genes

The lotus genome DNA data (Ming et al., 2013) was downloaded from COGE (v2, id16885, https://www.genomevolution.org/coge/) and gene prediction was performed with hidden Markov model (HMM) program (Eddy, 1998). Hidden Markov models of TPP and TPS domain were obtained from PFAM (http://pfam.xfam.org/). Motif scanning in TPS was done by PROSITE scan (Hulo et al., 2006). Gene structures of *TPS* genes were analyzed on the Gene Structure Display Server 2.0 (GSDS; http://gsds.cbi.pku.edu.cn/).

### Multiple sequence alignment and phylogenetic analysis

Multiple sequence alignments in full-length protein sequences were executed by using MUSCLE 3.52, and this was followed by manual refinement and comparisons (Edgar, 2004; Cao et al., 2014). Phylogenetic analysis was performed with MEGA 6.0 using a maximum likelihood method with the JTT (Jones, Taylor, and Thornton) amino acid substitution model. Bootstrap support values were estimated using 1000 pseudo-replicates.

### Protein properties and conserved motif analysis

Protein properties of *TPS* genes, e.g., the molecular weight (MW), isoelectric point (*p*I), and grand average of hydropathicity (GRAVY) were calculated using ProParam (http://web.expasy.org/protparam). Analysis for conserved motifs in TPS proteins was carried out using MEME (http://meme.sdsc.edu/meme/cgi-bin/meme.cgo) with the default parameters.

### Functional divergence analysis

To analyze the functional divergence between groups and predict the amino acid residues resulting in functional difference, program DIVERGE (Gu and Velden, 2002) was applied to calculate the coefficient of type I functional divergence θ_I_ as well as that of type II functional divergence θ_II_. The likelihood test is approximated to a χ^2^ distribution with 1 degree of freedom. After duplication, a significant θ_I_ means site-specific changed selective constraints (Xun, 2003; Gu, 2006; Yang et al., 2009). After duplication, a significant θ_II_, indicates amino acid physiochemical property's obvious shifts (Xun, 2003; Gu, 2006; Yang et al., 2009).

### Site-specific selection assessment and testing

The value of *d*_*N*_/*d*_*S*_ ratio (or ω, the ratio of non-synonymous to synonymous distances) for each group was analyzed with the program codeml from PAML version 4 (Yang, 2007). A likelihood ratio test (LRT) was used to detect variation in ω among sites between M0 vs. M3 and M7 vs. M8, respectively. The χ^2^ statistics with degrees of freedom equal to the differences between parameters is twice the log likelihood difference between the two models in the LRT (3 for M0/M3 tests and 2 for M7/M8 tests; Mondragon-Palomino and Gaut, 2005). If the LRT was statistically significant and the evolution of genes below groups, then sites are assumed to be under positive selection pressure through Bayes methods (Yang et al., 2005).

### *In silico* expression analysis

To identify expression pattern of *TPS* genes, expression level in different tissues and abiotic stress treatments, transcriptome datasets of lotus was analyzed. The data sets obtained from GenBank include: rhizome apical meristem (SRS413495), rhizome elongation zone (SRS413496), rhizome internode (SRS414455, SRS414456, SRS414457, SRS414458, SRS414459), leaf (SRS413488, SRS413489, SRS413490, SRS413491, SRS413492), petiole (SRS413498, SRS413499, SRS413500, SRS413501, SRS413502), root (SRS412272, SRS412273, SRS412278, SRS412279, SRS412280). The library preparation methods and statistics of sequencing data were detailed in Kim et al. (2013). To take advantage of our in house transcriptome data, we analyzed the expression pattern of *TPS* genes in response to submerged and 5 mm Copper-treated conditions. Three-month-old lotus seedlings were used for submergence (24 h) and 5 mm Copper-treatment (24 h). To achieve submergence, seedlings were transferred to a plastic tank and water was added into the tank to a water depth of 20 cm from top of lotus. The sample without copper or submergence treatment was the control (Con). After the treatments, total RNA was isolated using the CTAB-LiCl method. For mRNA-seq, three sets of total RNA were used for cDNA library construction and sequencing (without replicates) at the Beijing Genomics Institute (BGI, Shenzhen, China), following the manufacture's protocols.

All SRA format data were converted into FASTQ using the SRA toolkit. All raw reads were first filtered with NGS QC Toolkit (v2.3.3) to remove adapter sequences, reads containing poly-N, and low-quality sequences (*Q* < 20). An index of the lotus genome was built using Bowtie 2 and paired-end or single-end clean reads were aligned to the reference genome using TopHat. The expression levels (fragments per kb per million mapped read, FPKM) from the representative transcript were determined using cufflinks program (cufflinks v2.2.142) with default settings. We used a threshold of FPKM ≥ 1 to define a gene as “expressed.”

### Real-time quantitative PCR

Two month-old lotus seedlings were used for submergence treatment and copper treatment. Seedlings were completely submerged or treated with 5 mm copper for 24 h (as described above) and then rhizomes were sampled. The sample without copper or submergence treatment was the control (Con). Meanwhile, different tissues of control seedlings were sampled. Tissues were homogenized with mortar and pestle in liquid nitrogen. Total RNA was isolated using CTAB-LiCl method. About 4 μg of total RNA was reverse-transcribed using an oligo(dT) primer and SuperScript Reverse Transcriptase (Invitrogen, USA). Real-time quantitative PCR was conducted using SYBR green (TaKaRa Biotechnology) on Mastercycler ep realplex real-time PCR system (Eppendorf, Hamburg, Germany) with a final volume of 20 μl per reaction. The specific primers were listed in Table S1. The relative transcript abundance was normalized using lotus *Actin* gene. Gene expression in various tissues were represented by *NnTPS*/*Actin*. Fold changes of genes under copper stress and submergence treatment were values relative to control samples after normalization to *Actin* transcript levels. RT-qPCR data presented were the means ± SE of at least four independent experiments. Differences among treatments were analyzed by on-way ANOVA, taking *P* < 0.05 as significant according to multiple comparisons or *t*-test.

## Results and discussion

### Genome-wide identification of lotus *TPS* gene family members

Accumulating evidence showed that *TPS* genes play a vital role in modulating plants response to abiotic stresses, i.e., *OsTPS1* could improve rice-seed tolerance to low temperature, salt, and drought (Jiang et al., 2010; Vandesteene et al., 2010; Li et al., 2011; Zang et al., 2011). However, identity and function of lotus *TPS* gene family was previously undescribed. The lotus genome was sequenced recently, which makes it possible to carry out genome-wide study on *TPS* genes in lotus. Plant TPS proteins contain a TPS as well as a trehalose-6-phopshate phosphatase (TPP) domain (Yang et al., 2012). Thus, HMM profile of two conserved TPS domains (TPS and TPP domain) was used to search local lotus protein data with HMMER program (Sonnhammer et al., 1998; Ross et al., 2007). Nine lotus TPSs (NnTPSs) gene family members were identified in lotus genome (Table 1). Taking advantage of these identified genes, we further conducted a homology search from genome and amino acid sequences of lotus. However, the search did not yield new potentially TPS encoding genes for lotus beyond the nine initially identified. The identified NnTPSs contained a similar number of amino acid from 845 (NNU_024672) to 937 (NNU_016432). The isoelectric point (PI) ranged from 5.60 (NNU_022788) to 6.33 (NNU_004429). The protein weight ranged from 95.37 (NNU_024672) to 106.21 (NNU_016432) kDa. Nine NnTPS scattered in 7 scaffolds. Scaffold 4 and 5 had 2 *NnTPS* genes, respectively. Previous studies have identified 11 TPS in *Arabidopsis*, 11 in rice, and 12 in poplar (Yang et al., 2012), suggesting that the number of genes remains stable in this family and that usually duplicated copies of *TPS* genes are not retained after a whole-genome duplication event (Hao et al., 2014).

**Table 1:** **List of the *NnTPS* genes identified in this study**.

**Gene accession number**	**Scaffold ID**	**Predicted position**	**CDS (bp)**	**Deduced polypeptide**				**Predicted subcellular localization**
				**Length (aa)**	**MW (kDa)**	**pI**	**GRAVY**	
NNU_020889	Scaffold_3	36472545-36476497	2598	865	97.58	5.86	−0.195	Cytoplasmic
NNU_016707	Scaffold_5	12364436-12368019	2580	859	97.05	6.02	−0.143	Plasma membrane
NNU_024672	Scaffold_8	6741636-6749305	2535	845	95.37	5.72	−0.167	Cytoplasmic
NNU_014679	Scaffold_20	449424-453667	2604	867	97.60	6.10	−0.183	Cytoplasmic
NNU_020115	Scaffold_17	1846465-1854756	2616	871	97.98	5.64	−0.183	Cytoplasmic
NNU_004429	Scaffold_4	32965362-32971524	2586	861	97.13	6.33	−0.092	Plasma membrane
NNU_000253	Scaffold_4	13382224-13386331	2649	883	100.39	5.76	−0.187	Cytoplasmic
NNU_016432	Scaffold_5	37054048-37058407	2814	937	106.30	5.94	−0.155	Plasma membrane
NNU_022788	Scaffold_9	4294971-4298958	2547	848	96.35	5.60	−0.275	Cytoplasmic

### Gene structure analysis of TPS in lotus

**Figure 1:**
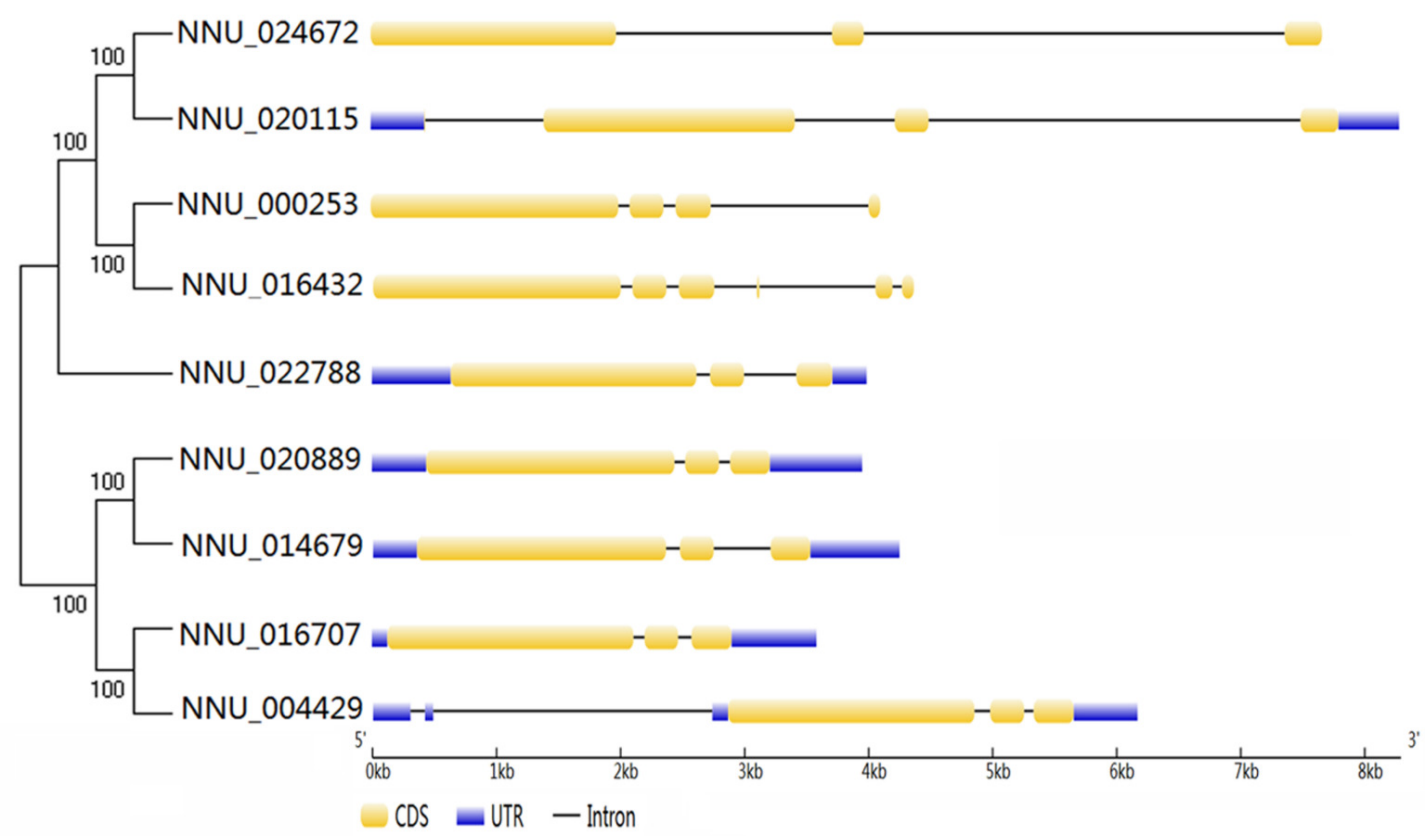
**Structure and phylogenetic analysis of NnTPS proteins**. The unrooted phylogenetic tree resulting from the full-length amino acid alignment of all the NnTPS proteins is shown on the left side of the figure. Exon-intron structures of the identified *NnTPS* genes are shown on the right side. The graphic representation of the optimized gene model displayed using GSDS.

To assess the evolutionary relationship of nine *NnTPS* genes, a small evolutionary tree was constructed using the nine *NnTPS* genes to exam *NnTPS* gene structures which are typical imprints of evolution within some gene families. The length of gene introns was larger and genetic structures were more complicated in NNU_024672, NNU_020115, NNU_000253, and NNU_016432 than the other *NnTPS* genes (Figure 1). All *NnTPS* genes contain at least one intron in their CDSs: most of them have three introns, one (NNU_004429) has four introns and one (NNU_022788) has five introns. According to the previous reports (Nuruzzaman et al., 2010; Hu et al., 2016), the rate of intron gain is slower than that of intron loss after segmental duplication in rice. Thus, it can be concluded that NNU_022788 might be the most ancestral with the other *NnTPS* genes derived from it. Compared with similar CDS length (2535–2814 bp) among 9 *NnTPS* genes, their gene length is even more variable (3583–8291 bp).

### Conserved sequences in TPS proteins

**Figure 2:**
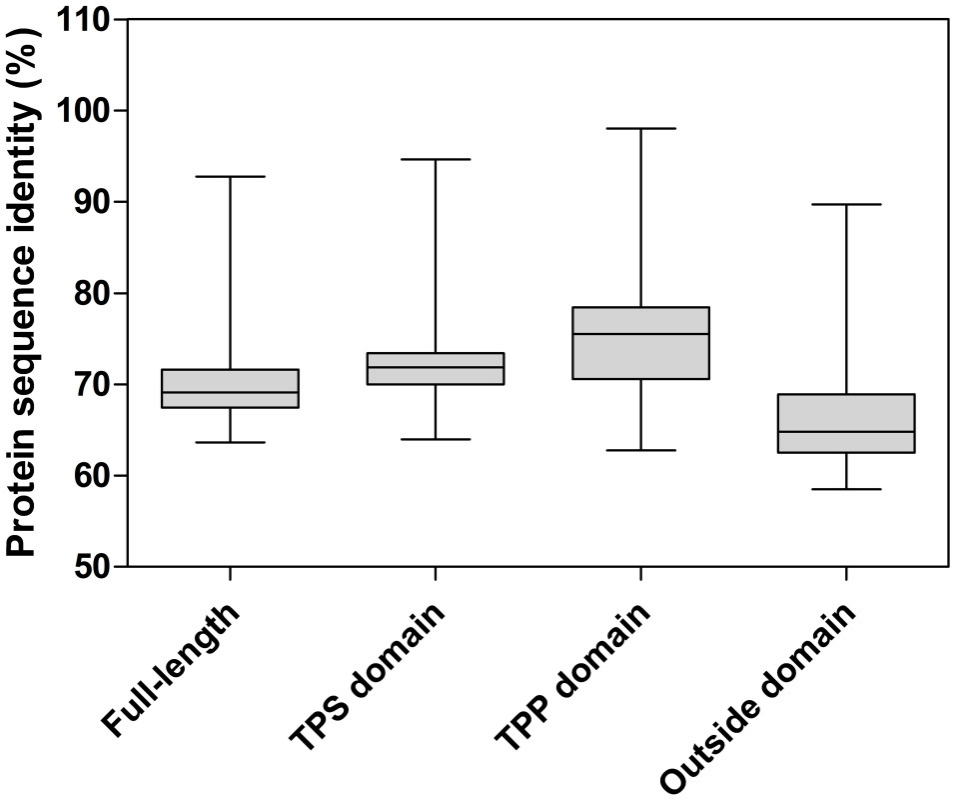
**Pairwise sequence identity of full-length TPS proteins**. Pairwise sequence identities between TPS domain, TPP domain, full length protein sequence, and sequence outside domain were calculated. The boxplot shows the median (black line), interquartile range (box), and maximum and minimum scores (whiskers) for each data set.

A homology analysis of nine full-length protein sequences indicated that nucleotide identity among them ranged from 63.66% (between NNU_016707 and NNU_022788) to 92.78% (between NNU_024672 and NNU_020115; Figure 2; Figure S1). Reflecting what was found in the protein sequences, a high similarity (ranging from 65.14 to 90.26%) was shared among nine *NnTPSs* full-length sequences. Several residues in the catalytic center were highly conserved in all *NnTPS* genes (Figure S1) suggested that corresponding genes encoding active enzymes. Nevertheless, relatively high divergence was observed in some regions of amino acid sequences outside of the domain. It is likely that these un-conserved regions might contribute to functional distinction.

Subcellular localization of NnTPS isoforms was predicted using web subcellular localization predictor server (http://cello.life.nctu.edu.tw/; Table 1). Translated proteins of NNU_016707, NNU_004429, and NNU_016432 were predicted as being located on plasma membrane while most of NnTPSs were located in cytoplasm.

**Figure 3:**
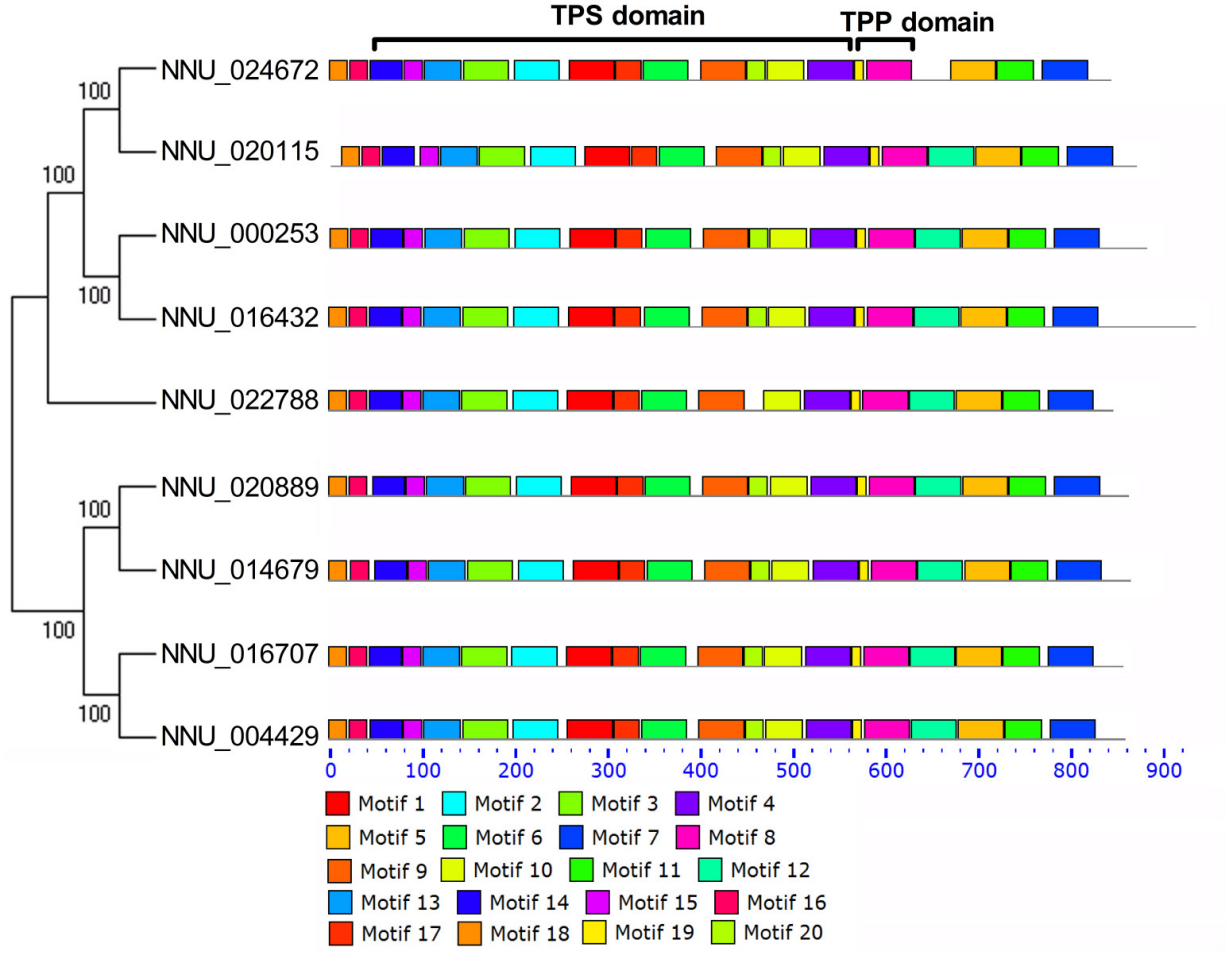
**Schematic diagram of amino acid motifs of TPS protein**. Motif analysis was performed using MEME 4.11.2 as described in the methods. The different-colored boxes represent different motifs and their position in each TPS sequence. The sequences of key motifs that located in TPS and TPP domain were indicated.

The identification of motifs for all NnTPS proteins were performed by software MEME (Bailey and Elkan, 1994) with default settings. We obtained sequences of 20 motifs and the distribution of these motifs in TPS proteins (Figure 3; Table S2). These motifs were conserved in all *NnTPS* genes except NNU_024672 which lacked motif12. According to Figure 3, motifs 1, 2, 3, 4, 6, 9, 10, 13, 14, 15, 17, and 20 were located in the TPS domain and motifs 8 and 19 were located in TPP domain. These features in conserved motifs of NnTPS were also observed in other plants (Mu et al., 2016).

**Figure 4:**
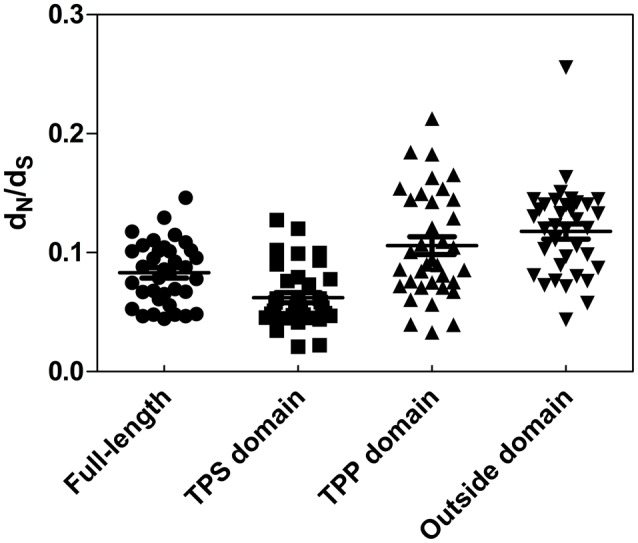
**The *d*_*N*_/*d*_*S*_ ratios for different regions of lotus TPS proteins**.

**Figure 5:**
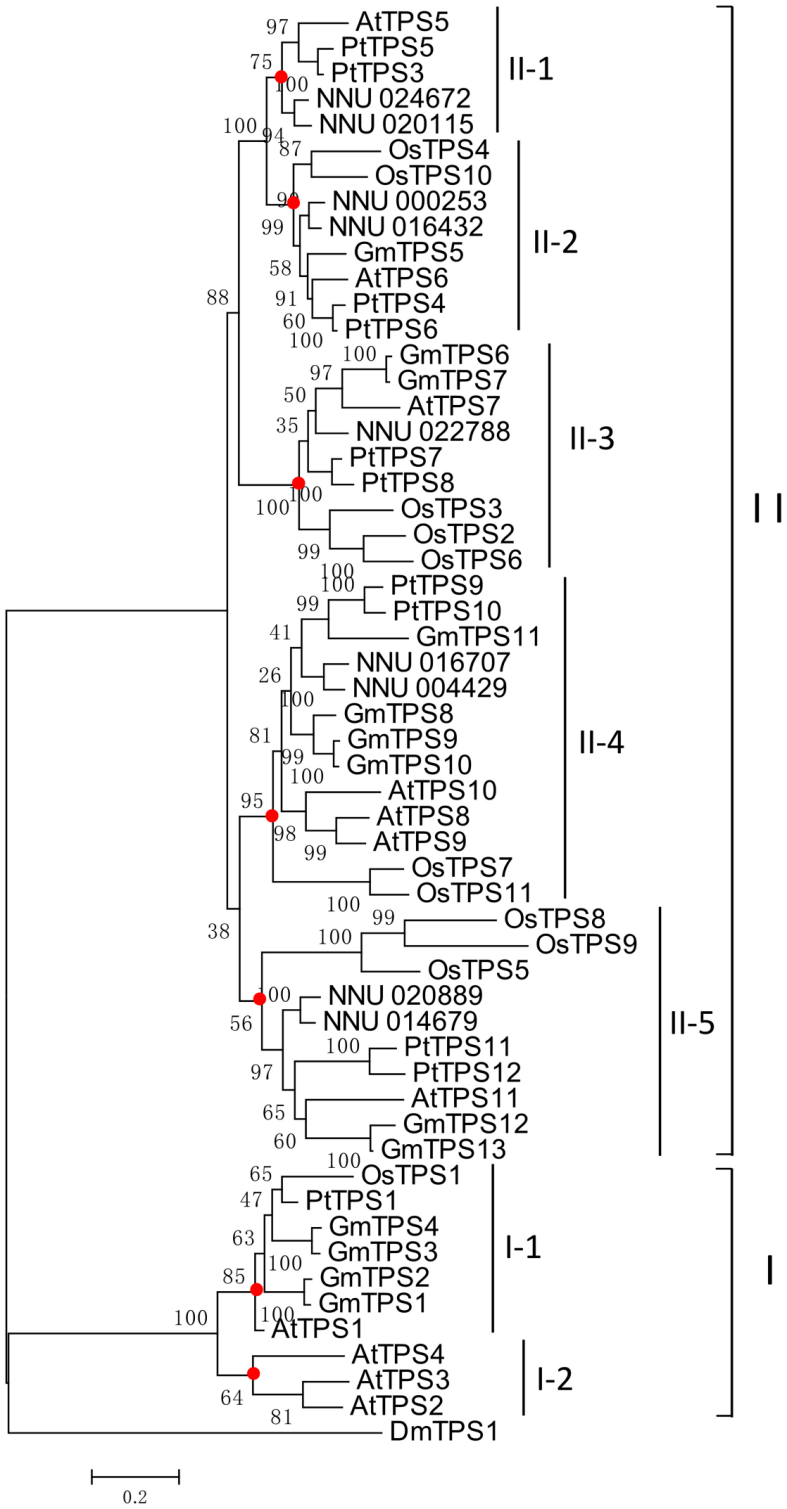
**Phylogenetic relationships of the TPS from lotus, Arabidopsis (AtTPS), poplar (PtTPS), soybean (GmTPS), and rice (OsTPS)**. Red circles indicate the most recent common ancestral *TPS* genes among dicots and rice.

To explore whether selection pressure had affected *NnTPS* genes, we calculated ratio (ω) of the synonymous substitution rate (d_*S*_) vs. the non-synonymous substitution rate (d_*N*_) using the program codeml from PAML (version 4). Generally, *d*_*N*_/*d*_*S*_ ratio >1 indicates positive selection, a ratio < 1 indicates negative or purifying selection and a ratio = 1 indicates neutral evolution (Wang et al., 2005; Song et al., 2015). Results in **Figure 5** showed that *d*_*N*_/*d*_*S*_ ratios were always less than 1 for different domains and regions outside domain, suggesting purifying selection on lotus *TPS* gene family. The *d*_*N*_/*d*_*S*_ ratios outside NnTPS domain were found to be much higher than the ratios inside the NnTPS domains (Figure 4). These results showed that NnTPS domains evolved faster than regions outside TPS domain. The relaxed purifying or positive selection in the regions outside TPS domain may lead to the above results.

### Relationships of lotus TPS family with other plant TPSs

A phylogenetic tree was used to reveal homologous relationships and evolutionary roots of TPS from *Arabidopsis*, rice, poplar (Yang et al., 2012), and soybean (Yang et al., 2012). The amino acid sequence alignment of TPS in these plants was conducted for phylogenetic tree construction using MEGA 5.1 (Figure 5). According to the phylogenetic tree and previous division of *Arabidopsis* and rice *TPS* genes, these *TPS* genes are divided into two subfamilies (I and II; Vandesteene et al., 2010; Zang et al., 2011). *Arabidopsis*, rice, soybean, and poplar contain four (*AtTPS1, 2, 3*, and *4*), one (*OsTPS1*), four (*GmTPS1, 2, 3*, and *4*), and one (*PtTPS1*) subfamily I TPS, respectively. However, no lotus TPS subfamily I members was found. It should be noted that the absence of subfamily I TPS in lotus might be due to incomplete genome sequencing coverage (86.5%). However, we cannot exclude the possibility that these genes have been fatally lost in evolution, as the number of *TPS* genes in subfamily I is far less than *TPS* genes in subfamily II in previous reported species (Zang et al., 2011). In order to describe paralogous and orthologous relations among this family, the subfamily II *TPS* genes were further divided into five group (II-1, 2, 3, 4, and 5) with high bootstrap support, suggesting that genes in each subgroup might share a similar origin. In most cases, subgroups are duplicates of at least one lineage. For example, subgroup II-2 and II-3 were duplicated after the separation between dicot and monocots. We identified nodes that lead to dicots (lotus, *Arabidopsis*, poplar, and soybeans)-specific and monocots (rice)-specific subfamilies. Group II-2 only contained lotus, *Arabidopsis*, and poplar Class II *TPS* genes, indicating that genes might have been lost from the rice genome. Subfamily I-1 only contained three *Arabidopsis* Class I TPS. Moreover, most proteins in lotus TPS family were contained in paralogous pairs, including 4 pairs of NnTPS accounting for 89% NnTPSs. This result indicated that most lotus *TPS* genes expanded in a species-specific manner.

Functional divergence of the duplicate genes has been recognized as an important source of evolutionary novelty (Yang et al., 2012). To evaluate potential functional divergence, type I and type II functional divergence between groups of the TPS family were estimated by posterior analysis (Gu and Velden, 2002). For this analysis, the collected plant TPS proteins were used and estimation was based on multiple alignments of proteins for any two groups (Table 2). Previous work proposed several possible evolutionary fates of duplicate genes (Hughes, 1994; Force et al., 1999). Our results showed that most type I coefficients (θ_I_) of functional divergence were significantly greater than zero (*P* < 0.01), while few of the type II coefficients (θ_II_) was statistically greater than zero. These results suggested that type I functional divergence was the dominant pattern for evolution of TPS family in these plants, and significantly site-specific altered selective constraints should contribute to most of the TPS members which would, after the diversification, cause a group-specific functional evolution.

**Table 2:** **Functional divergence between groups of the *TPS* gene family in plants**.

	**Θ_*I*_ ± SE^a^**	**Type I**		***Q_k_* ≥ 0.80^b^**	***Q_k_* ≥ 0.95^c^**	**Type II**	
		**LRT**	***P***			**Θ_*II*_ ± SE**	***P***
II-1/II-2	0.217 ± 0.101	4.607	0.032	1	0	−0.014 ± 0.072	0.846
II-1/II-3	0.352 ± 0.118	8.919	0.003	0	0	0.014 ± 0.08	0.861
II-1/II-4	0.444 ± 0.107	17.262	0.000	4	1	−0.019 ± 0.096	0.843
II-1/II-5	0.525 ± 0.127	16.947	0.000	6	0	−0.043 ± 0.107	0.688
II-1/I-1	0.57 ± 0.123	21.432	0.000	14	0	0.477 ± 0.066	0
II-2/II-3	0.143 ± 0.083	2.998	0.085	0	0	−0.002 ± 0.087	0.982
II-2/II-4	0.191 ± 0.075	6.425	0.011	1	0	−0.043 ± 0.102	0.673
II-2/II-5	0.266 ± 0.07	14.453	0.000	3	1	−0.04 ± 0.113	0.723
II-2/I-1	0.386 ± 0.092	17.500	0.000	6	0	0.425 ± 0.075	0
II-3/II-4	0.153 ± 0.075	4.162	0.041	1	0	−0.046 ± 0.106	0.664
II-3/II-5	0.281 ± 0.079	12.654	0.000	2	0	−0.066 ± 0.116	0.569
II-3/I-1	0.434 ± 0.089	24.043	0.000	6	1	0.487 ± 0.074	0
II-4/II-5	0.081 ± 0.047	2.979	0.085	0	0	−0.101 ± 0.125	0.419
II-4/I-1	0.304 ± 0.089	11.711	0.001	2	0	0.408 ± 0.088	0
II-5/I-1	0.415 ± 0.093	19.781	0.000	4	1	0.432 ± 0.093	0

a *The coefficient of functional divergence between two subgroups and its standard error*.

b *The number of critical amino acid residues with posterior probability (Q_k_) >0.80*.

c *The number of critical amino acid residues with posterior probability (Q_k_) >0.95*.

**Figure 6:**
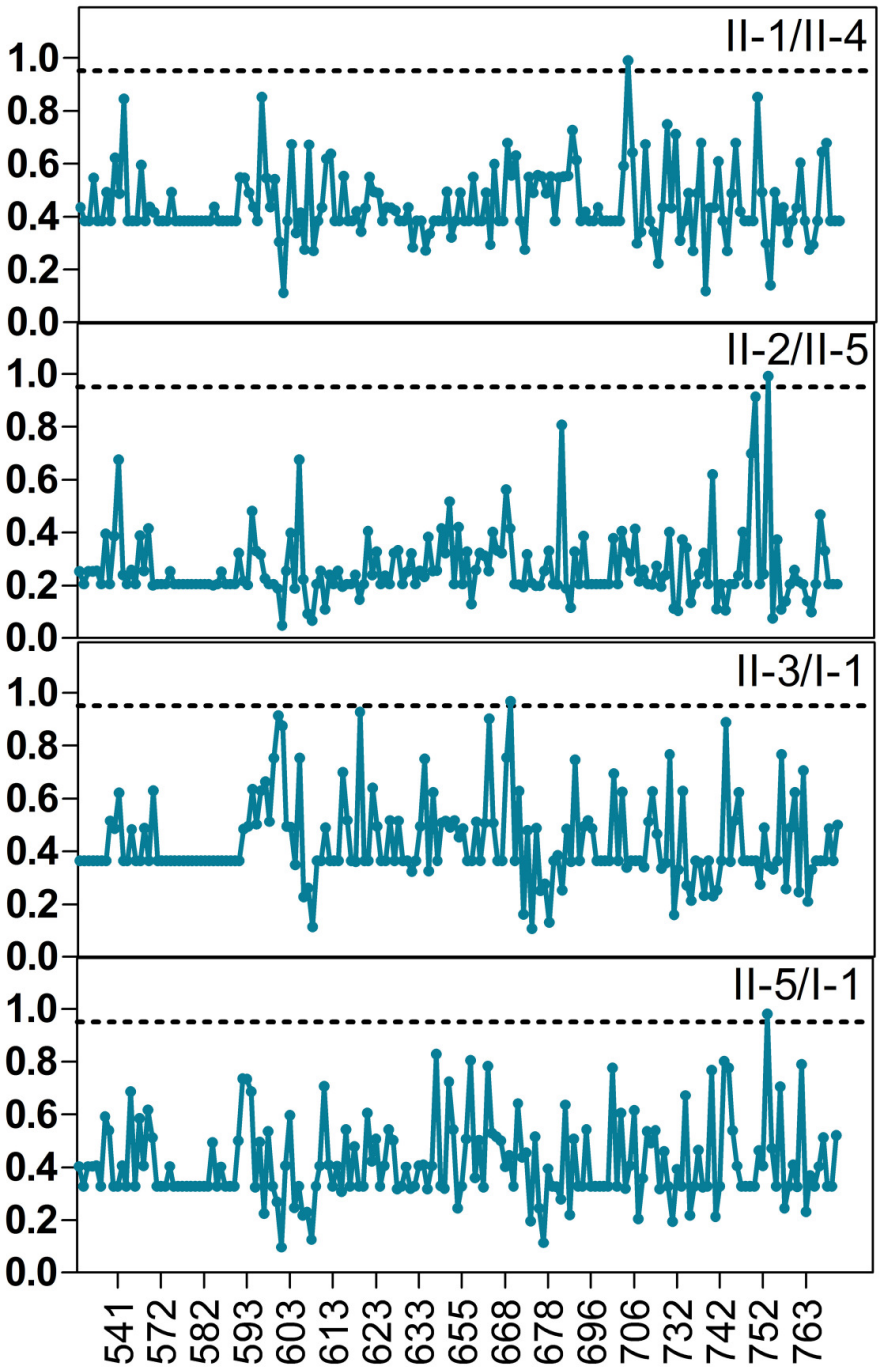
**Type I functional divergence among the plant *TPS* gene members**. Posterior probability (PP) profiles of the site-specific type I functional divergence. The line indicates cutoff = 0.95.

Further, the calculation of site-specific profiles on the basis of posterior analysis for all group pairs with functional divergence would predict the critical amino acid residues, which are accountable for the functional divergence. Among aligned sites, most of them had low posterior probabilities. *Q*_*k*_ ≥ 0.80 and *Q*_*k*_ ≥ 0.95 were utilized empirically as cutoffs to determine type I functional divergence-related residues existing between groups, so as to greatly reduce false positive (Wang et al., 2011). For most of pairs of groups, at least one site had the posterior probability higher than 0.8, and four pairs of groups had one site with posterior probability higher than 0.95 (Figure 6). A cut-off value Q(k) ≥ 0.95 was used for estimated key amino acid residues. The number of amino acid residues that presumably contributed to alter functional constraints was small between TPS groups. Three amino acid residues (669, 704, and 753) in all comparisons were identified as being most important for the functional divergence. These amino acids are all localized in the TPP domain.

To examine if selective pressure between the seven groups of *TPSs* genes would change, the likelihood ratio tests of positive selection were used via the ML methods as well as codon substitution models (Yang, 1998; Yang and Bielawski, 2000; Yang et al., 2012). Two tests were performed during the analysis process. First, models M0 and M3 were compared in order to assess whether *d*_*N*_/*d*_*S*_ ratio variations existed among codon positions below each group. On the whole, the maximum likelihood estimates of *d*_*N*_/*d*_*S*_ values as for groups under model M0 were close to zero, with three exceptions of *Arabidopsis TPS* genes in group I-2. This implied that the principal force which acting on the evolution of TPS family was purifying selection (Table 3). It is obvious that although the majority of protein residues are heavily dependent on constant purifying selection, some sites are also being impacted by positive selection. Positive selection is an important adaption mechanism; thus, sites under positive selection pressure may have sped up functional divergence of TPS; in this case it would allow the plants to adjust to its environment. The results we get are in accordance with this conclusion. As the statistical differences of log likelihood between model M0 and M3 were significant for all groups, it suggests that the overall selective constraint levels varied across the TPS family group lineages. Secondly, the LRT which is applied to compare data fit to models M7 vs. M8 was used to determine whether positive selection promoted divergence of TPS family below groups or not. Two groups (group I-1 and II-2) of the 7 groups analyzed, were believed to have undergone positive selection, because they satisfied that (1) an estimate of ω > 1 under M8, (2) sites identified to be under positive selection, and (3) a significant LRT. This result suggests that positive selection contributed to the evolution of genes in these groups. When the LRT suggested positive selective action had occurred, positively selected sites are identified under model M8 using Bayesian method (Nielsen and Yang, 1998; Yang and Bielawski, 2000). We found 31 and 4 positively selected sites in subgroup I-1 and II-2, respectively.

**Table 3:** **Detection of positive selection under site-specific model**.

**Group**	**N^a^**	***d_N_*/*d_S_*(ω) under M0^b^**	**2Δ/M3 vs. M0^c^**	**2Δ/M8 vs. M7^c^**	**M8 estimates^d^**	**Positively selected sites^e^**
I-1	7	0.140	620.331^**^	123.550^**^	*p*1 = 0.117, ω = 1.833	31
					β (*p* = 1.000, *q* = 10.380)	
I-2	3	0.425	72.153^**^	16.600^**^	*p*1 = 0.031, ω = 19.426	–
					β (*p* = 0.428, q = 0.560)	
II-1	5	0.095	145.763^**^	1.774	*p*1 = 0.018, ω = 1.328	–
					β (*p* = 0.333, *q* = 2.962)	
II-2	8	0.079	610.610^**^	32.373^**^	*p*1 = 0.019, ω = 217.11	4
					β (*p* = 0.345, *q* = 3.406)	
II-3	9	0.104	609.630^**^	0.008	*p*1 = 0.00001, ω = 3.705	–
					β (*p* = 0.389, *q* = 2.518)	
II-4	13	0.149	981.750^**^	7.786^**^	*p*1 = 0.015, ω = 1.263	–
					β (*p* = 0.511, *q* = 2.624)	
II-5	10	0.164	852.843^**^	0.621	*p*1 = 0.002, ω = 4.772	–
					β (*p* = 0.532, *q* = 2.021)	

a *Number of the sequences in the group*.

b *d_N_/d_S_ (ω) is the average ratio over sites under a codon model with one-ratio*.

c *Double asterisks represent P < 0.01 for chi-square (χ^2^) test, while single asterisk represents P < 0.05*.

d *ω is estimated under model M8; p1 is the inferred proportion of positively selected sites; p and q are the parameters of the beta distribution*.

e *The amino acid, which is listed in positively selected position, had a posterior probability > 0.95 under M8*.

### Gene expression analysis

**Figure 7:**
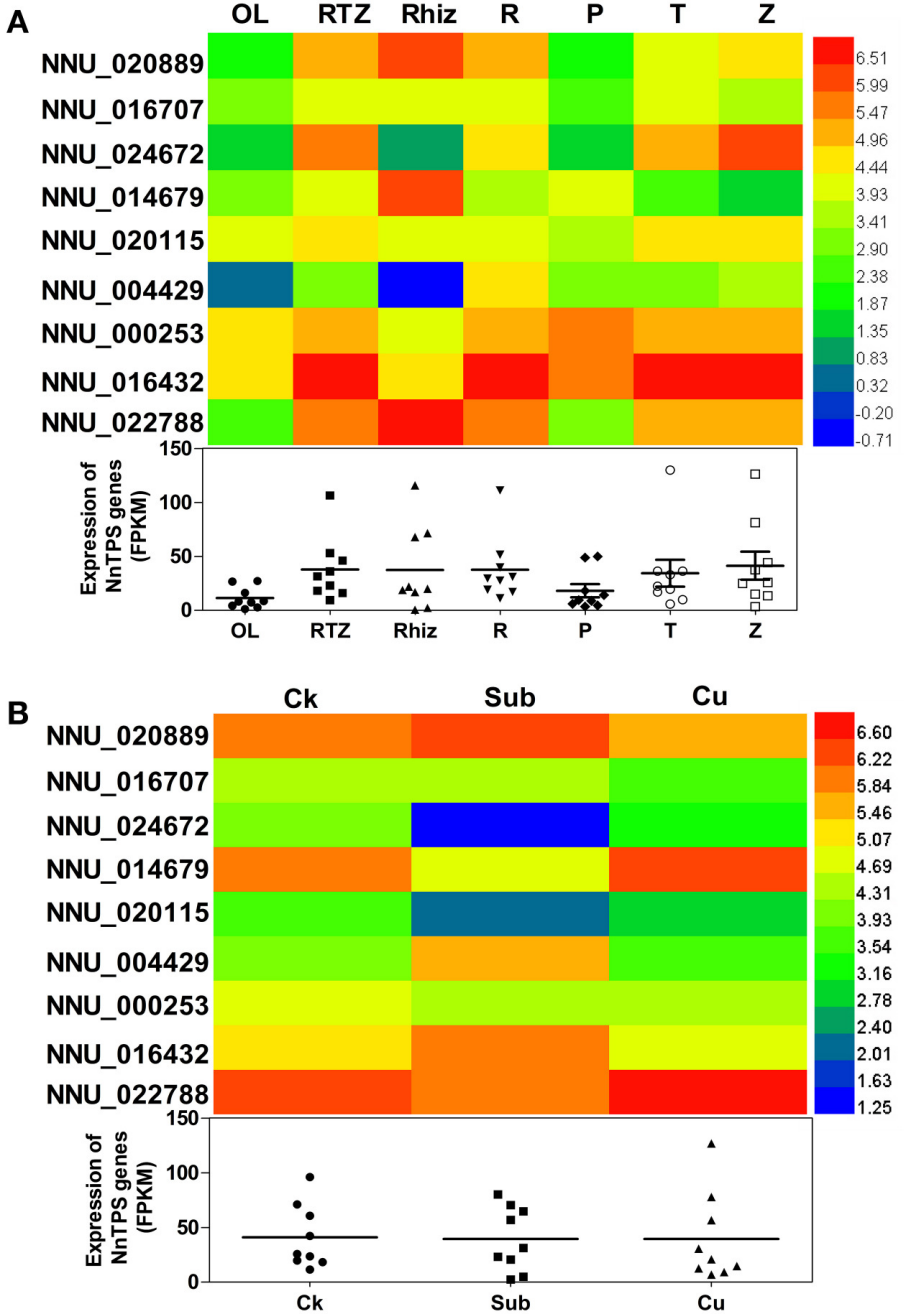
**Expression profiles of the nine *NnTPS* genes in different tissues (A) and upon submergence and copper stresses (B)**. The color scale represents RPKM normalized log_2_ transformed data. Red indicates high expression level and blue indicate low expression level. OL, leaf; RTZ, Combined Rhiz Tip Zone; Rhiz, Rhizome internode; R, Root; P, Petiole; T, Rhizome apical tip; Z, Rhizome elongation zone.

*TPS* genes are known to be involved in development and stress responses. In this study, expression pattern of *TPS* genes in different lotus tissues has been studied using transcriptome data set of lotus (available at NCBI; Kim et al., 2013) as well as our in house transcriptome dataset of lotus under submergence and copper stress. *NnTPS* genes showed differential expression level in various lotus tissues (Figure 7A). No tissue-specific expressed *NnTPS* genes were found. Among them, NNU_016432 showed the highest expression level while NNU_004429 showed the lowest transcript level in most examined tissues, in comparisons with other *NnTPS* genes. Genes from the same group frequently showed similar expression pattern within specific tissues. For example, both NNU_000253 and NNU_016432 had relatively low expression in leaf and rhizome internode, and high expression in root, rhizome apical tip, and rhizome elongation zone. Based on FPKM calculations, the total transcript abundance of *NnTPS* genes were obviously low in leaf and petiole and high in root and rhizome. These results indicated that *NnTPS* genes might primarily have functions in sink tissues.

In response to submergence and copper stress, the total transcript abundance of NnTPS did not show apparent difference (Figure 7B). Two *NnTPS* genes (NNU_0044229 and NNU_016432) were highly induced by submergence treatment. In contrast, most *NnTPS* genes (i.e., NNU_024672, NNU_014679, and NNU_022788) were down-regulated. The abundance of NNU_024672 decreased over 80%. Submergence leads to lower concentration of oxygen in a plant as a result of a lower diffusion rate of molecular oxygen in water as opposed to air, which would ultimately influence energy metabolism. The reaction of NNU_024672 to low oxygen demonstrates possible involvement in energy metabolism in the seedlings and that highly expressed *NnTPS* genes could play vital roles during this complicated biological process. Some plant metabolic processes contain copper as an essential micronutrient, functioning as a constituent of enzymes and other proteins, and in photosynthetic reactions (Maksymiec, 1998; Hall, 2002; Yruela, 2009; Martins et al., 2014). Excessive copper can lead to oxidative stress and be phytotoxic (Mithöfer et al., 2004). It can inhibit growth, influence plant metabolism and affect the function of several enzymes (Martins and Mourato, 2006; Mourato et al., 2009). Under copper stress, the transcript abundance of most *NnTPS* genes was not obviously changed, except two *NnTPS* genes (NNU_014679 and NNU_022788). It is interesting that abundance of two genes were apparently decreased in response to submergence while increased upon copper stress. We speculate that under copper stress more energy was needed to strengthen antioxidant system, while more energy was needed to be preserved under submergence as photosynthesis is inhibited. The contrasting expression pattern under submergence and copper stress indicated that NNU_014679 and NNU_022788 might play important roles in lotus energy metabolism and participate in stress response. Similar to previous work, TPS has been reported to play an important role in the regulation of carbohydrate metabolism and especially the perception of carbohydrate availability (Müller et al., 2001).

**Figure 8:**
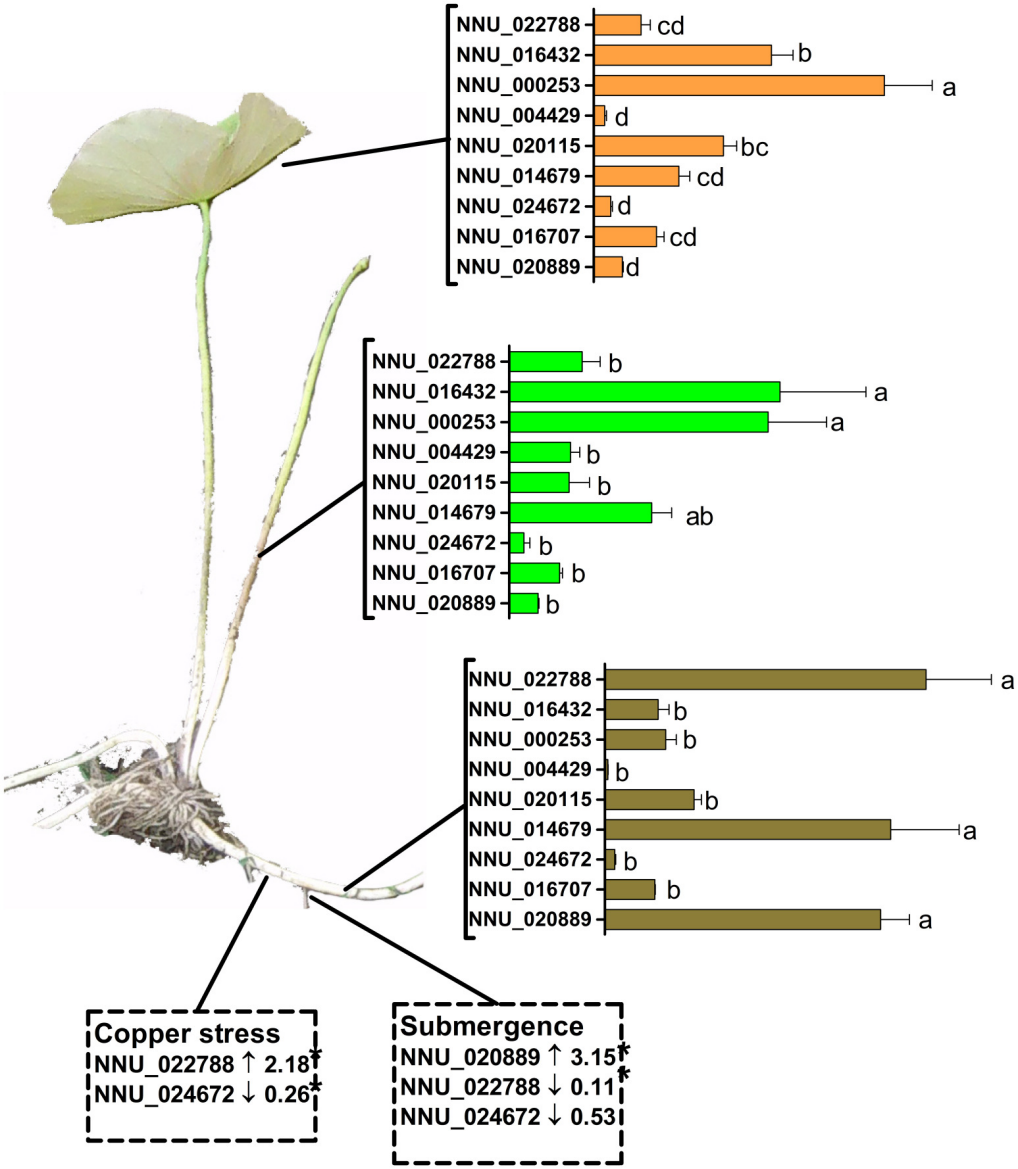
**Validation of expression of selected *NnTPS* genes in various tissues and in rhizome upon exogenous stimuli**. Tissues including leaf, petiole, and rhizome were sampled from 2 month old lotus seedlings. Meanwhile, lotus seedlings were treated with submergence stress or copper stress for 24 h and then rhizome was sampled. The sample without 5 mm copper treatment or submergence treatment was the control (Con). Gene expression was analyzed by real-time RT-PCR. The relative transcript abundance was normalized using lotus actin gene. Values are means ± SE of at least four independent experiments. Bars with different letters are significantly different at *P* < 0.05 according to Turkey's multiple range test. Fold changes of genes under copper stress and submergence treatment were values relative to control samples. Asterisks indicate significant differences compared with control sample at *P* < 0.05 levels according to *t*-test.

*In silico* analysis showed that *NnTPS* genes exhibited differential abundance in various tissues and some of them are obviously regulated by environmental stimuli. Some of these obviously changed genes were then validated by RT-qPCR. As shown in Figure 8, RT-qPCR results of genes under various tissues and stresses were similar in magnitude to those obtained by deep sequencing. NNU_020889, NNU_014679, and NNU_022788 were the most abundant genes in rhizome. It is interesting, that while under submergence, NNU_020889 was significantly induced while NNU_000788 was significantly down-regulated. Moreover, NNU_024672, which has the minimum transcript level were further down-regulated upon submergence.

## Conclusion

Collectively, our results characterize the structure, evolutionary, and some functional features of the lotus *TPS* gene family. Based on systematic analysis of lotus genomes, we identified 9 *TPS* genes. Gene structure analysis showed that NNU_022788 might be the ancestral member, from which other genes were derived. Regions outside the TPS domain in nine *NnTPS* genes evolved faster than regions within the TPS domains. Based on phylogenetic tree constructed using *TPS* genes from lotus, *Arabidopsis*, polar, soybean, and rice, *TPS* genes could be classified into two main subfamilies (I-II). Lotus *TPS* genes showed species-specific expanded manner. Three amino acid residues (669, 704, and 753) in TPP domain were identified as the most important residues for the functional divergence of these *TPS* genes. Moreover, 2 groups (group I-1 and II-2) were believed to have undergone positive selection based on likelihood ratio test. Expression pattern analysis showed that *NnTPS* genes might mainly have function in sink tissues and two *NnTPS* genes (including NNU_014679 and NNU_022788) might play important roles in lotus energy metabolism and stress responses.

## Author contributions

QJ and XH conceived the study; QJ, BW, and XL did data analysis and drafted the manuscript; NM, YW, HJ, and YX revised the manuscript. All authors read and approved the final manuscript.

## Funding

This work was supported by Funding of agricultural science and technology innovation of Jiangsu Province, China (CX(15)1030), National Natural Science Foundation of China (31501795, 31400600), the China Postdoctoral Science Foundation funded project (2014M560432, 2015T80563), the Natural Science Foundation of Jiangsu Province in China (BK20151229, BK20140695), and the fundamental research funds for the central universities (KJQN201659).

### Conflict of interest statement

The authors declare that the research was conducted in the absence of any commercial or financial relationships that could be construed as a potential conflict of interest.

## Supplementary material

Figure S1 **Amino acid sequence alignment of three lotus TPS proteins**. Strictly conserved sequence is in white on black background; similar amino acids are in black on green background. Residues involved in the catalytic center are placed in boxes.

Table S1 **List of primers used in this study**.

Table S2 **Detail information of identified motifs**.
