# Supplementary figures and images for: Genome-Wide Identification and Evolution Analysis of Trehalose-6-Phosphate Synthase Gene Family in Nelumbo nucifera
Source: Front Plant Sci. 2016 Sep 29;7:1445. doi: 10.3389/fpls.2016.01445 (PMC5040708; doi:10.3389/fpls.2016.01445)

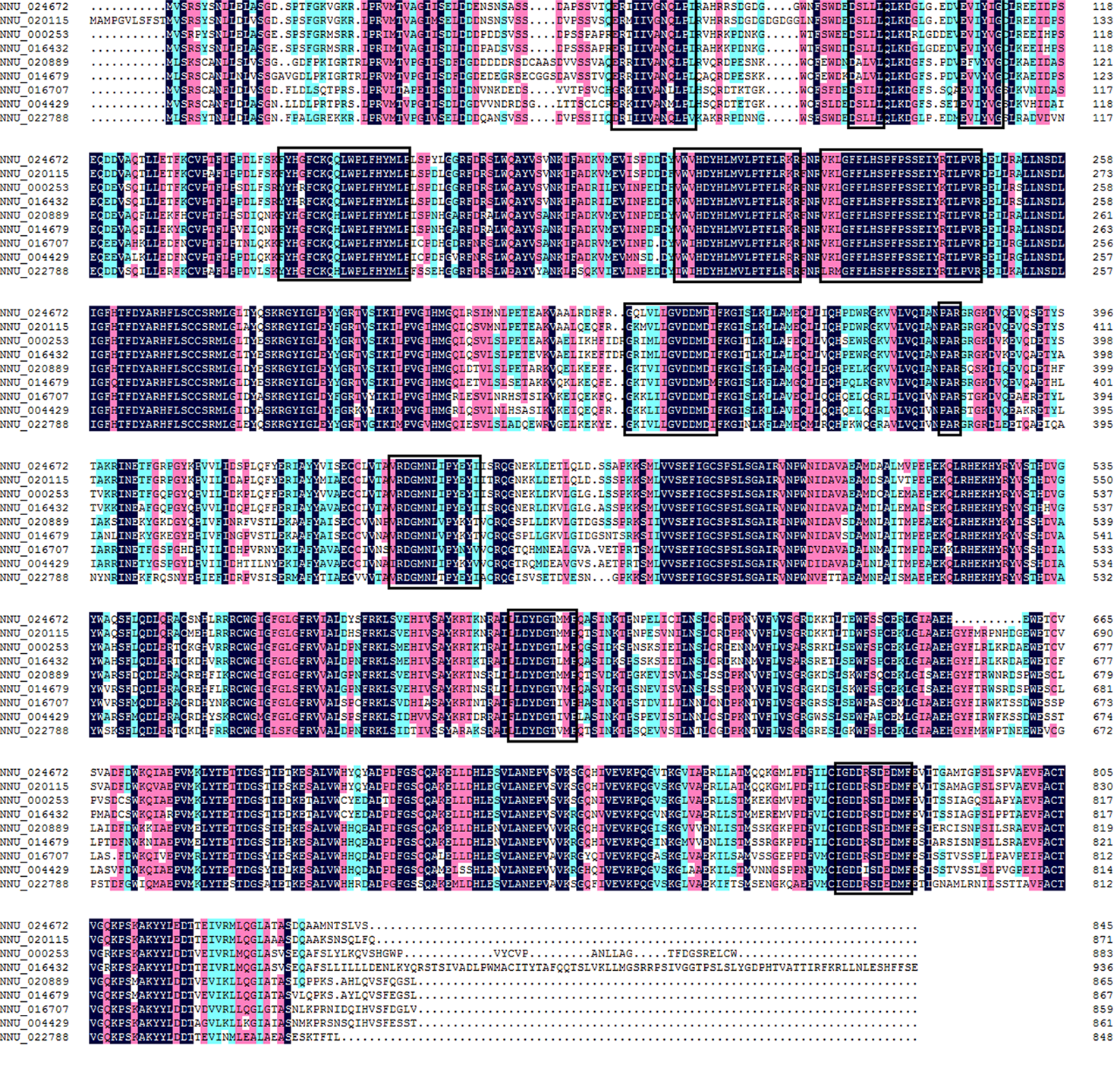

Supplement: Figure S1 — Amino acid sequence alignment of three lotus TPS proteins. Strictly conserved sequence is in white on black background; similar amino acids are in black on green background. Residues involved in the catalytic center are placed in boxes. [file Image1.TIF]
