# Supplementary material for: Genome-Wide Identification and Evolution Analysis of Trehalose-6-Phosphate Synthase Gene Family in Nelumbo nucifera
Source: Front Plant Sci. 2016 Sep 29;7:1445. doi: 10.3389/fpls.2016.01445 (PMC5040708; doi:10.3389/fpls.2016.01445)
Supplement: Table S1 — List of primers used in this study. [file Table1.DOCX]

**Table S1** List of primers used in this study.

| Primer name | Forward sequence (5’→3’) | Reverse sequence (5’→3’) |
| --- | --- | --- |
| NNU_020889 | TGCCAATCTCGCCTAACC | AACCCAGACGTAATCCTCAT |
| NNU_016707 | AGCCAGAAGGATCAACGA | TTCCGCCACAGCATAAAA |
| NNU_024672 | TTCTCCGAGCCCTCCTAA | TGTCCATGTCATCCACCC |
| NNU_014679 | TTTCGTCGGGAGCGGTAG | CGGCGAGAACCCATCTTT |
| NNU_020115 | TTCTCCGAGCACTCCTAA | CAAAGTATTCAAGCCCAAT |
| NNU_004429 | AGGGAGTAAGTAAAGGGTT | GCAGTATCATCCAGGTAG |
| NNU_000253 | AGATAACAAGGGCTGGAC | GGTAGTATCGGCTAAATAGG |
| NNU_016432 | TGAAGTATCAGCGGAGTA | GAATCTTATTGTGGTGGC |
| NNU_022788 | TTGCGTGTAGACAGGGAATC | AAGCCACATCATGGGTGC |
| Actin | AAGAAGAAGAAGAAGAAGA | CATACAAGAGGCTGAATA |
